# Advances in Enhanced Menaquinone-7 Production From *Bacillus subtilis*

**DOI:** 10.3389/fbioe.2021.695526

**Published:** 2021-07-19

**Authors:** Chaoyong Liao, Hammed Ayansola, Yanbo Ma, Koichi Ito, Yuming Guo, Bingkun Zhang

**Affiliations:** ^1^ State Key Laboratory of Animal Nutrition, Department of Animal Nutrition and Feed Science, College of Animal Science and Technology, China Agricultural University, Beijing, China; ^2^ Henan International Joint Laboratory of Animal Welfare and Health Breeding, Department of Animal Physiology, College of Animal Science and Technology, Henan University of Science and Technology, Luoyang, China; ^3^ Department of Food and Physiological Models, Graduate School of Agricultural and Life Sciences, The University of Tokyo, Ibaraki, Japan

**Keywords:** *Bacillus* subtilis, menaquinone-7, synthetic biology, chassis cells, CRISPR interference, CRISPR activation

## Abstract

The production of nutraceutical compounds through biosynthetic approaches has received considerable attention in recent years. For example, Menaquinone-7 (MK-7), a sub-type of Vitamin K2, biosynthesized from *Bacillus subtilis* (*B. subtilis*), proved to be more efficiently produced than the conventional chemical synthesis techniques. This is possible due to the development of *B. subtilis* as a chassis cell during the biosynthesis stages. Hence, it is imperative to provide insights on the *B. subtilis* membrane permeability modifications, biofilm reactors, and fermentation optimization as advanced techniques relevant to MK-7 production. Although the traditional gene-editing method of homologous recombination improves the biosynthetic pathway, CRISPR-Cas9 could potentially resolve the drawbacks of traditional genome editing techniques. For these reasons, future studies should explore the applications of CRISPRi (CRISPR interference) and CRISPRa (CRISPR activation) system gene-editing tools in the MK-7 anabolism pathway.

## Introduction

Vitamin K (VK) occurs in its natural forms, such as Phylloquinone (VK1) and menaquinone (MK; VK2), and synthetic form as menadione (VK3). Leafy vegetable contains the highest concentration of VK1 (Widhalm et al., 2012), whereas bacteria synthesize VK2. The VK2 structure comprises a naphthoquinone ring with an isoprenoid side chain–depending on the isoprenoid numbers (4–13). On this basis, VK2 can be in different forms, such as MK-4∼MK-13 (Bentley and Meganathan, 1982).

**FIGURE 1:**
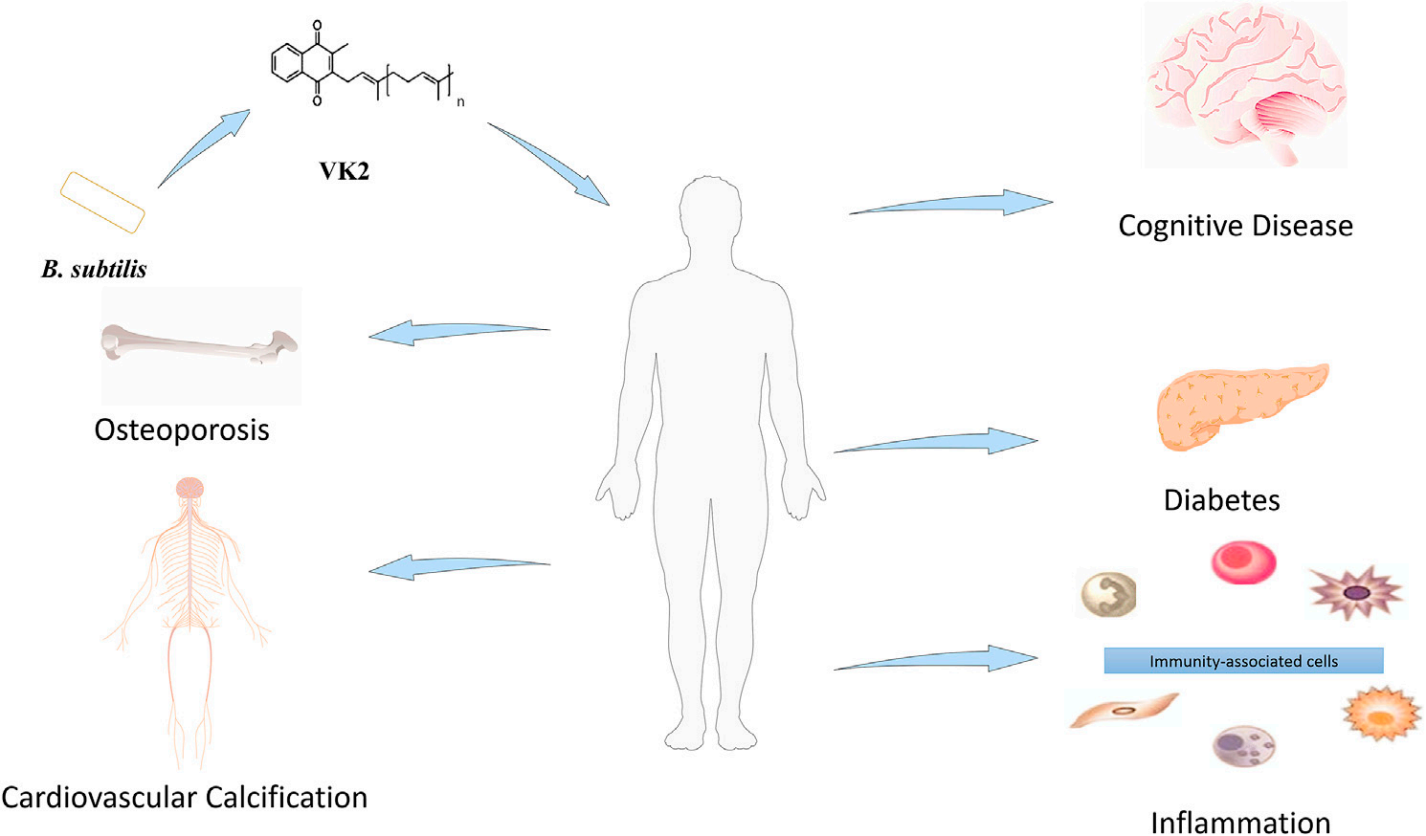
Positive effects of VK2 in multiple disorders.

VK2 confers beneficial effects in osteoporosis (Capozzi et al., 2019), cardiovascular calcification (Fernández et al., 2014), cognitive disease (Tanprasertsuk et al., 2020), inflammation (Ohsaki et al., 2010), and diabetes (Manna and Kalita, 2016) (Figure 1). Besides, VK-dependent proteins (VKDPs) are associated with bone and vascular systems in humans (Fusaro et al., 2011). Osteocalcin (OCN), a VKDP, participates in bone mineralization (Lanham et al., 2015). Structurally, VK2 is biologically active only in its trans-isomeric form, while the chemically synthesized cis-isomeric form is biologically inactive. On this basis, it is challenging to synthesize a stereoselective biologically active all-trans configuration of VK2 chemically. Therefore, trans-isomeric VK2 is currently in high demand. VK2 microbial production is comparatively preferred because it can selectively produce all-trans isomers; however, faced with low yield. The challenge now is to develop new techniques that will boost the bacterially synthesized VK2 at industrial scales (Table 1). In line with this objective, this review highlights the relevance of advanced biotechnologies applicable to VK2 production.

**TABLE 1:** VK2-producing bacteria and fermentation method to improve VK2 production.

Bacteria type	VK2 type	Concentration (mg/L)	Fermentation techniques for optimization of MK-7 production	References
*Flavobacterium* sp. 238-7-k3-15	MK-4	155	An addition of cedarwood oil in a detergent supplemented culture medium	Tani and Taguchi (1989)
	MK-6	27		
*Lactic acid bacteria*	MK-7; MK-8	0.123	The strains are grown either in reconstituted non-fat dry milk or a soymilk medium	Morishita et al. (1999)
	MK-9; MK-10			
*B. subtilis* BCRC 14715	MK-7	7.8	Modification of the functional constituents has a significant effect on the fermentation process and the black soybean between (40–45)°C optimized MK-7 yield	Wu and Chou (2009)
*Bacillus amyloliquefaciens*	MK-7	11.7 ± 0.6	The addition of 4% glycerol enhances MK-7 yields at 43°C in the cheonggukjang fermentation medium	Wu and Ahn (2011)
KCTC11712BP	MK-4	0.8		
*Bacillus subtilis* natto	MK-7	62.3	Centralized composite face design optimized nutrient substrate at 5% yeast extract; 18.9% soy peptone; 5% glycerol and 0.06% K_2_HPO_4_	Berenjian et al. (2011)
*Bacillus subtilis* natto	MK-7	140	Mathematical model predicts the optimal environmental conditions (temperature and water activities) to optimize MK-7 yield in solid-state fermentation bioreactor	Mahanama et al. (2012)
*Bacillus subtilis* natto	MK-7	40.96	Surfactants regulate *B. subtilis* membrane permeability for maximized MK-7 yield	Hu et al. (2017)
*Bacillus subtilis* natto (NF1)	MK-7	14.7 ± 1.4	The biofilm reactor also can promote the MK-7 content of *B. subtilis* natto fermented with glycerol, yeast extract, and soytone	Mahdinia et al. (2018b)
*Bacillus subtilis* 168	MK-7	360	The dual-function phr60-rap60-spo0A quorum sensing system is used to dynamically control the synthesis of MK-7	Cui et al. (2019)
*Bacillus subtilis* 168	MK-7	69.5 ± 2.8	The modular metabolic engineering designs enhance MK-7 biosynthesis when *B. subtilis* 168 is used as the chassis	Yang et al. (2019)
*Bacillus subtilis* 168	MK-7	50	The rate-limiting enzymes (dxs, dxr, idi, and MenA) are over-expressed in different combinations to genetically modify the *B. subtilis* 168 strains used for the fermentation processes	Ma et al. (2019)
*Bacillus subtilis* BS20	MK-7	415	The five genes of the MEP pathway are over-expressed in *Bacillus subtilis* BS20	Chen et al. (2020)
*Bacillus subtilis* 168	MK-7	281.4 ± 5.0	The recombinant strain BSMK11 with simultaneous overexpressing the *glpK, glpD, aroGfbr, pyrGfbr, hepS, vgb* genes*,* and knockouting the *mgsA* and *araM* genes	Yang et al. (2020)
*Bacillus subtilis* 168	MK-7	310	The oxalate decarboxylase OxdC has an important effect on electron generation and MK-7 synthesis, when the transcriptional level of NADH dehydrogenase decreases in static culture	Cui et al. (2020)

*B. subtilis,* a Gram-positive bacterium, is predominantly present in the soil. It is used in molecular microbiology due to its ideal genetic architecture (Mohan et al., 2020). However, the primary reason for the broad application of *B. subtilis* is based on its prominent characteristics, such as 1) lack of pathogenicity and endotoxin, making it to be generally recognized as safe (GRAS), 2) high growth rate (widely used as a cell factory to produce pyridoxine and enzymes) (Commichau et al., 2014; Zafar et al., 2014; Yadav et al., 2020), and 3) relative ease in genetic manipulation (for large-scale industrial production) (Nicolas et al., 2012; Mars et al., 2015; Koo et al., 2017). Moreover, *B. subtilis* can produce a wide range of VK2 isoforms, whereby MK-7 accounts for over 90% of the total output (Sato et al., 2001). For these reasons, *B. subtilis* remains the preferred host for MK-7 production. In order to further optimize *B. subtilis* strains’ production capacity, there has been increasing interest in the genetic engineering of their biochemical pathways in recent years. Hence, there is a need to explore this new Frontier to produce bacterially synthesized compounds efficiently.

Menaquinones (MKs), including MK-7, are lipid-soluble compounds that play crucial roles in electron transport activities during metabolism. Particularly in microbial cells, they transfer electrons between the membrane-bound protein complexes in the electron transport chain (Meganathan, 2001). For instance, studies have previously reported various forms of menaquinones involved in bacterial fermentation, such as MK-8 in *Escherichia coli* (Koch et al., 1971), MK-9 in *Corynebacterium diphteriae* (Scholes and King, 1965), and MK-7 in *B. subtilis* (Farrand and Taber, 1974). In *B. subtilis*, MK-7 acts as an intermediate molecule in the electron transport chain (Abul-Hajj, 1978) and is involved in the coupling of ATP synthesis (Kunst et al., 1997). MK-7 is also required for glycosylation of specific membrane proteins and the early stages of sporulation (Farrand and Taber, 1974). Therefore, MK-7 biosynthesis is integral to *B. subtilis* survivability.

*B. subtilis* is an ideal genetically modified engineering bacteria and has a strong development significance due to its physiological advantages of GRAS, rapid growth and clear genetic background for genetic manipulation. As well as MK-7 biosynthesis is crucial for the survival of *B. subtilis*. Among the VK2 subtypes, MK-7 is the most effective and has the highest half-life of 68 h in the circulatory system. Likewise, it has more biological functions than other Vitamin K family members (Schurgers et al., 2007). Therefore, it is pertinent to describe the methods involved in optimizing the *B. subtilis* MK-7 expression system.

## Methods to Improve Menaquinone-7 Production From *B. subtilis*

The conventional mode of bacterial MK-7 yield enhancement is mutation breeding. Mutation breeding has been adopted as the conventional means to enhance the yield of MK-7 during bacterial biosynthesis. This technique selectively modifies microorganisms under experimental conditions, including physical or chemical methods, to induce genetic mutations (Che et al., 2018). Several factors such as the time of ultraviolet irradiation, type, dosage, and treatment duration of the mutagenic reagent determine the induction of genetic mutations in microbes. However, the main drawback of this method is difficulty to screen positive mutants to obtain effective results. For these reasons, mutation breeding is gradually being replaced significantly by molecular and other efficient approaches. On this premise, the subsequent sections will address novel techniques relevant to increasing MK-7 production effectively from *B. subtilis.*

## Alteration of *B. subtilis* Membrane Permeability

Surfactants (SAAs) are compounds that reduce the solvent surface tension and promote hydrophobic solute solubility in water. The chemical structure of the SAA comprises a polar hydrophilic and non-polar lipophilic group. In this phenomenon, the surfactants form a molecular layer between their interface by disrupting the fluidity and integrity of the phospholipid molecules within the cell membrane as well as the interaction between the phospholipid molecule and the membrane integrin (Cortez and Roberto, 2012). Common examples of SAA include the anionic surfactants-consisting hydrophobic groups (alkyl chains of various lengths, alkyl phenyl ethers, and alkylbenzenes) and the hydrophilic group (carboxyl, sulfuric acid, sulfonate, phosphate) such as sodium dodecyl sulfate (SDS), sodium lauryl sulfate, and ammonium dodecyl sulfate. These ionic surfactants denature membrane proteins by forming channels within the cell membrane and interferes with cell membrane synthesis (Bansal-Mutalik and Gaikar, 2003). The hydrophobic solutes are categorized into four forms based on the hydrophilic charge after molecular dissociations. These comprise: 1) common cationic SAAs, including dehydroabietylamine (DA), 2) dodecyltrimethylammonium bromide (DTA), 3) cetyltrimethylammonium bromide (CTAB), and 4) triethanolamine (TEA). On the other hand, the non-ionic surfactants are Tween-60, Tween-80, APG, TritonX-100, PO-10, and POE. With these different forms, adequate SAAs in the culture medium improve the intracellular metabolite extraction and decrease the metabolite accumulation in the cell in previous studies.

Note, SAA increases membrane permeability but consequently destroys the fluidity and integrity of cell membranes, which will affect the MK-7 and *MenA* proteins located on the cell membrane. Therefore, a suitable SAA should not only reduce the negative feedback mechanism of MK-7 but also ensure the normal physiological function of the cell membrane. For this reason, researchers should consider the timing and specific SAA to use for optimizing MK-7 yield from *B. subtilis*. “Tween 80”, an effective stimulant for some bacteria (Zhang et al., 2006) and fungi (Zhang and Cheung, 2011), is a efficient SAA to achieve these objectives. Likewise, the non-ionic surfactant “span 20” has been demonstrated to increase MK-7 production in *B. subtilis* natto (Hu et al., 2017).

## Biofilm Reactor for Menaquinone-7 Production

The microbial species and the surface environment determine the biofilm structure, chemistry, and physiology (Kuchma and O’Toole, 2000). A biofilm reactor system promotes microbial migration from the planktonic state to form biofilms (Mahdinia et al., 2017). As a result, biofilms can obtain ideal products using appropriate microbes (Demirci et al., 2007; Ercan and Demirci, 2013). In line with this phenomenon, recent investigations show that biofilm reactors could enhance the production of numerous microbial metabolites, such as biofuels, enzymes, and biopolymers (Ho et al., 1997; Khiyami et al., 2006; Izmirlioglu and Demirci, 2016). Similarly, compounds such as amine-functionalized nanoparticles are more biologically compatible and stable than naked particles (Ranmadugala et al., 2017). This approach is accomplished using l-lysine coated 3-aminopropyl triethoxy silane, which introduces amine functional groups to increase nanoparticle binding to anionic cell membranes without affecting MK-7 production (Ebrahiminezhad et al., 2012; Alireza et al., 2015). However, APTES coated nanoparticles provide more anti-oxidative protection to nanoparticles crystal structure than l-lysine (Ghasemi et al., 2015).

The reports from a previous study demonstrate that batch feeding in *B. subtilis* culture significantly improves biological metabolites production (Mahdinia et al., 2018a). Subsequent studies also show that biofilm metabolism is better when the fermentation process is carried out with glucose. Besides, fermentation batches supplement with glycerol enhanced the metabolism in microbial cells, resulting in a higher MK-7 yield (Mahdinia et al., 2019). Therefore, MK-7 biosynthesis can be improved using the batch feeding method containing a carbon source (glucose or glycerol). The biofilm reactor also can promote the MK-7 content of *B. subtilis* natto fermented with glycerol, yeast extract, and soytone (Mahdinia et al., 2018b). Evidently, biofilm reactors present a reliable strategy to address the operational issues that occur during MK-7 biosynthesis on an industrial scale production.

The culture medium supplements with ions@APTES are reported as a promising carrier that changes the biofilm state to enhance the MK-7 secretion without affecting bacterial growth and viability (Ranmadugala et al., 2017). Mahanama et al. (2012) employe the improved Gompertz and the Luedeking-piret model to study the MK-7 biosynthesis in the biofilm reactor. The Luedeking-piret model efficiently increased MK-7 biosynthesis in the mixed model pattern containing both media and carbon sources. As such, biofilm reactors present a reliable strategy to address the operational issues during MK-7 biosynthesis on an industrial scale.

It is worth noting that MK-7 production by *B. subtilis* natto is associated with heavy pellicle and biofilm formation, which can cause operational issues in static fermentation (Stragier et al., 1988). However, these formations contribute to and almost constitute most of the MK-7 biosynthesis. This is the reason why the production of MK-7 has been confined to static fermentation practices but the conversion to large-scale stirred bioreactors is hindered. In biofilm reactors, mature biofilm structures exist and can withstand relatively high agitation and aeration rates, which render them a promising technology alternative to old static or suspended-cell bioreactors for MK-7 production (Mahdinia et al., 2017). Thus, the application of biofilm reactors in future studies could facilitate robust extracellular MK-7 secretion and present a reliable strategy to address the operational issues during MK-7 biosynthesis in industrial production.

## Optimization of Fermentation Process for Menaquinone-7 Extraction From Culture Medium

The amount of MK-7 extracted from the culture medium containing a mixture of n-butanol: n-hexane does not meet its full potential because menaquinone as MK-7 is a tightly bound membrane of bacterial cells (Minnikin et al., 1984). The non-disruptive extraction of MK-7 with pure organic solvents is inefficient, primarily, cytomembrane destruction facilitates the MK-7 extraction via ultrasonication, freeze-thawing, steam explosion, acid-heating, alkaline hydrolysis, and homogenization (Luo et al., 2016). For these reasons, it is suggested that optimizing the ratio of organic agents (such as n-butanol: n-hexane) could potentially improve MK-7 production in the culture medium. CaCl_2_ supplemented medium improved MK-7 extraction by implementing the salting-out method (Ahmed and Mahmoud, 2015). Another study shows that the mixture of n-butanol and n-hexane depleted MK-7 yield over time compared to control without n-butanol and ln-hexane. Such that the viability of *B. subtilis* cells significantly decreased after 60 h of periodic extraction with a mixture of n-butanol: n-hexane (1:2, v/v) (Ranmadugala et al., 2018). This reduced MK-7 production could result from poor biocompatibility of n-butanol and n-hexane mixture with *B. subtilis*. Also, n-butanol toxicity in the culture medium could be traced to n-butanol in the fermentation medium, thereby inhibiting the expansion of bacterial cells (Huang et al., 2010).

Apart from n-hexane, none of the other common vitamin K extraction solvents could maintain the viability of *B. subtilis* upon periodic extraction of MK-7. Most solvent mixtures containing a polar and non-polar solvent, such as the alcohol in the extraction solvent, could prevent the growth of bacteria by damaging the cell membrane (Ingram, 1976). In line with this context, it is reported that periodic extraction with n-hexane does not have any detrimental effect on the *B. subtilis* viability until 84 h. For example, MK-7 production (52.34 mg/L) is significantly optimized by n-hexane alone as a solvent for *B. subtilis* fermentation at 1.7∼fold higher than the control medium (Ranmadugala et al., 2018). The use of n-hexane in the fermentation medium may significantly increase the total yield of MK-7 within a short period compared to non-extracting control conditions. Another advantage of n-hexane as a solvent is its non-toxic effect in bacterial cells. For this reason, n-hexane can be an ideal extraction solvent for MK-7 with the ability to enhance biosynthesis productivity. And Fang et al. (2019) use ethanol to extract MK- seven twice directly from the crude cells after fermentation produced a MK-7 yield of 1.47 mg/g. The crude extract was purified using macroporous resin and subsequent crystallization. After treatment with HPD722 resin, the purification of MK-7 are 7.17∼fold increased with recovery yields of 97.2%. In general, n-hexane can be selected to extract the MK-7, then the purity of MK-7 is increased using macroporous resin followed by crystallization. The developed procedure can produce high-purity of MK-7 as well as high recoveries. This is an economical, efficient and simple method for preparing high-purity MK-7 on a large scale.

## Development of *B. subtilis* as a Chassis

Synthetic biology has presented the concept of genetic engineering, the standardization of living matter, the gene expression system, and its encoded proteins as biological building blocks. It is, therefore, imperative to understand synthetic biological terminologies, such as the optimization, transformation, or redesigning of biological components known as element engineering. Moreover, the designed cells using model organisms and then functionally re-engineered are called chassis cells. Notably, the chassis cells serve as the host for synthetic biological reactions.

Due to cellular complexity, embedded synthetic devices or systems may be affected by the cell’s original unrelated metabolic processes. The solution is to delete some non-essential genes to simplify the genome without affecting its function. One of the benefits of this strategy is that it reduces other metabolic processing energy and material requirements in the cell. In practice, the chassis cells are often modified as per the requirement, which entails embedding, replacing, or deleting genes or gene modules. Standard chassis cells include microbial cells, such as *E. coli, B. subtilis, yeast*, *Pseudomonas, and Mycoplasma* (Yan et al., 2018; Jores et al., 2019; Zhuang and Qi, 2019; Liang et al., 2020; Liu et al., 2020). For example, the deletion of 581.9–814.4 non-essential gene sequence of *B. subtilis* by gene degeneration method mitigated the guanosine and thymidine production (Li et al., 2016; Liu et al., 2018a; Cui et al., 2018). The MK-7 metabolic pathway gene optimization provides a theoretical basis for the MK-7 production using *B. subtilis* as a chassis. Usually, *B. subtilis* 168 is used as the chassis in modular metabolic engineering to promote the MK-7 biosynthesis (Ma et al., 2019; Yang et al., 2019). Therefore, the construction of *B. subtilis* as a chassis for MK-7 synthesis can provide a significant potential for industrial MK-7 production.

Constructing a chassis cell for efficient synthesis of MK-7 in *B. subtilis* requires two steps. In the first step, it is crucial to understand the MK-7 synthesis pathway in the bacteria. The reason is that chassis cells produce a high yielding strain after transforming the vector bacteria. For this reason, the chassis method thus resolves the fundamental challenge (i.e., synthetic pathway optimization) associated with the conventional technique used in MK-7 production, as discussed earlier. The second step of chassis cell construction focuses on the regulation of MK-7 synthesis-related gene expressions. This objective could be achieved by gene upregulation or branched gene knockout without affecting the bacterial physiological activities. For instance, overexpression of the MK-7 synthetic pathway gene and knockout of the synthetic branch gene *dhbB*, on the one hand, reduced the intermediate metabolite (isochorismate) consumption and, on the other hand, increased the carbon flux of the pathway for bacteria to synthesize MK-7, thereby promotes MK-7 production (Yang et al., 2019). The common practice in gene expression alteration is via homologous recombination of antibiotic resistance and anti-selective markers. Antibiotic resistance markers are laborious and time-consuming gene-editing techniques. However, the advent of CRISPR-Cas9 systems has helped scientists overcome these difficulties about homologous recombination and are also applicable as DNA binding tools with strong transformation ability.

## Microbial Biosynthesis of VK2

### VK2 Synthesis Pathway in Bacteria

**FIGURE 2:**
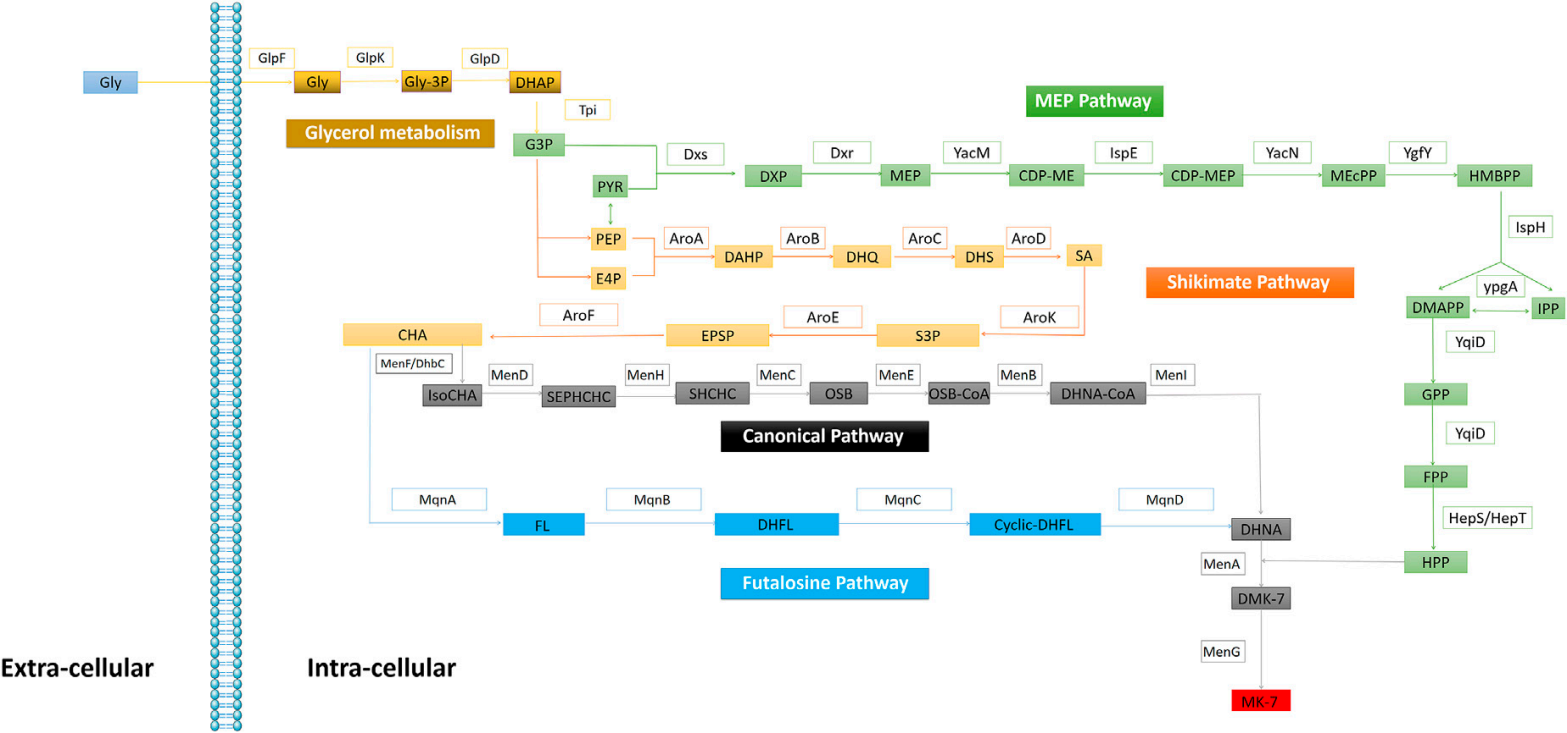
Enzymes in the *Bacteria* VK2 biosynthesis. Glycerol metabolism: GlpF (glycerol uptake facilitator), GlpK (glycerol kinase), GlpD (glycerol-3-phosphatedehydrogenase), Tpi (triosephosphate isomerase). MEP pathway**:** Dxs (1-deoxyxylulose-5- phosphate synthase), Dxr (1-deoxyxylulose-5-phosphate reductoisomerase), YacM (2-C-methylerythritol 4-phosphate cytidylyltransferase), IspE (4-diphosphocytidy-2-C-methylerythritol kinase), YacN (2-C-methylery thritol 2,4-cyclodiphosphate synthase), YgfY (4-hydroxy-3-methylbut-2-enyldiphosphate synthase), IspH (4-hydroxy-3-methylbut-2-enyldiphosphate reductase), YpgA (isopentenyl-diphosphate δ-isomerase), YqiD (farnesyl diphosphate synthase), HepS/HepT (heptaprenyl diphosphate synthase component I/II). Shikimate pathway: AroA (3-deoxy-7-phosphoheptulonate synthase), AroB (3-dehydroquinate synthase), AroC (3-dehydroquinate dehydratase), AroD (shikimate dehydrog enase), AroK (shikimate kinase), AroE (3-phosphoshikimate 1-carboxyvinyltransferase), AroF (chorismate synthase). Canonical pathway: MenF/DhbC (isochorismate synthase), MenD (2-succinyl-5-enolpyruvyl-6-hydroxy-3-cyclohexene-1-carboxylate synthase), MenH (2-succinyl-6 -hydroxy-2,4-cyclohexadiene-1-carboxylate synthase), MenC (*o*-succinylbenzoate synthase), MenE (*o*-succinylbenzoic acid-CoA ligase), MenB (1,4-dihydroxy-2-naphthoyl-CoA synthase); MenI (1,4-dihydroxy-2-naphthoyl-CoA hydrolase of *E. coli*), MenA (1,4-dihydroxy-2-naphthoate hepta prenyltransferase); MenG (demethylmenaquinone methyltransferase). Futalosine Pathway: MqnA (chorismate dehydratase), MqnB (futalosine hydrolase), MqnC (dehypoxanthine futalosine cyclase), MqnD (5,8-Dihydroxy-2-naphthoate synthase).

In 1982, Bentley elucidated the microbial VK_2_(MK-n; *n* = 4–13) biosynthesis pathway (Bentley and Meganathan, 1982). VK_2_ consists of naphthoquinone rings and isoprene side chains. In 2001, Meganathan further detail the methylerythritol (MEP) pathway, shikimate (SA) pathway (Meganathan, 2001), and the canonical pathway of VK_2_ biosynthesis in *B. subtilis* and *E. coli*. In addition to the canonical MK biosynthesis pathway, menaquinone is also synthesized via the futalosine pathway in *Streptomyces* (Shimizu et al., 2018) (Figure 2). Particularly in *B. subtilis* MK-7 synthesis, *Dxs* and *Dxr* are identified as the rate-limiting enzymes (Yuan et al., 2006; Lv et al., 2013); however, ypgA as a rate-limiting enzyme remains controversial (Julsing et al., 2007; Zhou et al., 2013).

## Gene Editing Tools for *B. subtilis* Via Homologous Recombination

The advance in genetic engineering has led to the development of effective strategies for *B. subtilis* gene-editing. *B. subtilis* is a widely used host for the production of enzymes and chemical compounds (Commichau et al., 2014; Zafar et al., 2014; Yadav et al., 2020). Improved genetic engineering strategies have turned *B. subtilis* into a highly efficient microbial cell factory for MK-7 production, and conferred comparative advantage over conventional industrial microbes, such as *E. coli* and *S. cerevisiae*. Optimizing the MK-7 biosynthetic pathway is an effective way to improve MK-7 production. It is commonly attained through the overexpression or deletion of the MK-7 metabolic pathway genes and optimization of biosynthesis pathway via homologous recombination using antibiotic-resistance and counter-selectable markers (Ma et al., 2019; Yang et al., 2019).

## Antibiotic-Resistance Markers

Antibiotic-resistance markers facilitate the insertion of antibiotic resistance genes into the host genomic DNA through homologous recombination. Integrons, which introduce antibiotic resistance markers into the genome to alter the host cell physiological state, can also be screened using antibiotic-resistance markers (Dempwolff et al., 2020). Unfortunately, the removal of resistance markers from the original genome in the integration vector is the problem of this technique (Biswas et al., 1993).

## Counter-Selectable Markers

Counter-selectable markers kill the microorganisms containing counter-selectable marker genes under some specific conditions (Reyrat et al., 1998). Counter-selectable markers can be utilized to eliminate antibiotic resistance genes integrated into the genome in a two-step process (Pritchett et al., 2004). Firstly, antibiotic resistance markers screen the integrons that contain integrated antibiotic resistance genes, target fragments, and counter-selectable marker genes incorporated into the genome. Secondly, the counter-selectable marker genes are screened to complete the seamless editing of the genome. Uracil phosphoribosyl transferase gene *upp* has been used as a counter-selectable marker of *B. subtilis* (Fabret et al., 2002). Besides, *araR*, which encodes a negative regulator of ara operon and is triggered by the l-arabinose enzyme of *B. subtilis,* is also used as a counter-selectable marker *B. subtilis* (Sa-Nogueira and Mota, 1997).

## Traditional Optimization Methods of Menaquinone-7 Synthesis Pathway in *B. subtilis*

In a previous study, increasing the carbon flux of the MK-7 synthesis pathway can increase the content of MK-7. Yang et al. (2019) over-express the *GlpD*, *MenA*, *Dxs*, *Dxr*, and *YacM-YacN* genes, as well deleted *dhbB* gene to enhance the MK-7 biosynthesis through glycerol metabolism. Ma et al. (2019) have shown that MK-7 production in *B. subtilis*168 could be successfully enhanced by stimulating the MEP-related rate-limiting genes’ co-expression and optimizing the gene sequence in the gene cluster. The optimal gene combination of *menA-dxs-dxr-ypgA* increases the MK-7 production by 11-fold (50 mg/L) than the standard vector. Moreover, Chen et al. (2020) have increased the production of MK-7 from *B. subtilis* BS20MEP by overexpressing 5 genes of the MEP pathway. Cui et al. (2019) use the dual-function *Phr60-Rap60-Spo0A* quorum sensing system to dynamically control the synthesis of MK-7 in the *B. subtilis* 168, that makes MK-7 output is increased by 40 times. Yang et al. (2020) obtain a recombinant strain BSMK11 modified to simultaneously overexpressing the *glpK, glpD, aroG* ^*fbr*^ *, pyrG* ^*fbr*^ *, hepS, vgb* genes, and knock-outing the *mgsA* and *araM* genes to increase the MK-7 production by 54.7%. The study also finds that oxalate decarboxylase OxdC has an important effect on electron generation and MK-7 synthesis, when the transcriptional level of NADH dehydrogenase decreases in static culture (Cui et al., 2020). It provides new ideas for the subsequent genetic modification of MK-7 strains.

## Future Perspectives on Gene Editing Tools for *B. subtilis*

Several standardized techniques are currently designed to regulate the *B. subtilis* gene expression (Popp et al., 2017; Yang et al., 2017). For instance, the dual-promoter system (Rao et al., 2020), the auto-inducible expression systems (Sun et al., 2020), and the highly active secretory expression system (Kang et al., 2020) are common genetic engineering used for *B. subtilis* gene modifications. These gene expression systems without markers have limited capability, which the CRISPR-Cas9 system can resolve. There are several databases containing information on *B. subtilis* DNA sequence, regulators, and metabolites in recent years. CRISPR-Cas9 system can explore this information to delete, mutate or insert genes at any desired locus position. Because of this mechanism, the CRISPR-Cas9 system has become the gamechanger in *B. subtilis* genome editing (Tobias et al., 2018; Yi et al., 2018; Lim and Choi, 2019; Watzlawick and Altenbuchner, 2019).

## CRISPR-Cas9 System Application for *B. subtilis*

Gene-editing tools based on the CRISPR system are widely used in synthetic biology. Currently, CRISPR-Cas9 is a hotspot technique for investigating and reducing off-target effects and multi-gene editing errors. Zuo et al. (2019) devise a method known as GOTI, a new off-target detection technology using CRISPR-Cas9, to test the gene-editing efficiency when off-target mutations are concerned. Their protocol helpes develop the emerging single-base editing technology known as GOTI, which can identify unpredictable off-targets, thereby improving gene-editing efficiency. For instance, Zhou et al. (2019) report that engineered deaminase can eliminate off-targeting effects. More so, Choi et al. (2019) also design a high-throughput platform by integrating mutation and systematic screening of *Streptococcus pyogenes* Cas9 protein to obtain the Opti-SpCas9. The high-throughput technique improved the editing precision without losing potency in a wide range of targets. In terms of gene-editing, Liu et al. (2018b) design the ReS-CuES system for rapid and efficient chromosomal rearrangement screening in distinct strains. Likewise, Zhang et al. (2019) devise a fast multiplex gRNA-tRNA array CRISPR-Cas9 (GTR-CRISPR) gene-editing system for *Saccharomyces cerevisiae*.

In *B. subtilis* CRISPR-Cas9 gene-editing, to overcome the problem of the instability of plasmid, low transformation efficiency, the introduction of the metabolic burden on the host cell, and counterselection methods. In recent years, some studies on CRISPR kits suitable for *B. subtilis* gene editing, such as Westbrook et al. (2016) insert the first gRNA cassette into a multiple gRNA delivery vector to construct a multiple gRNA delivery vector and clone each additional gRNA into a single gRNA delivery vector. Then, all gRNA delivery vectors are linearized and used to transform *B. subtilis*, the efficiency of single-gene mutation is 100%, and the efficiency of double gene mutation is 85%. Altenbuchner et al. (2016) use CRISPR-Cas9 vector to introduce two large deletions in the *B. subtilis 168s* chromosome. The single-plasmid system constructes for the genome editing of *B. subtilis* overcomes the problems of counterselection methods. Tobias et al. (2018) create a toolkit for simple and fast genetic engineering of phages recruiting *B. subtilis* as host system. This toolkit comprises the application CutSPR, a bioinformatic tool for rapid primer design, and facilitates the cloning of specific CRISPR-Cas9-based mutagenesis plasmids, and demonstrates reliability and high efficiency. García-Moyano et al. (2020) develop a vector compatible with high-throughput fragment exchange (FX) cloning for heterologous expression in *E. coli* and *Bacillus*. This vector catalog is through this work supplement with editing plasmid for genome engineering in *Bacillus* by adapting two CRISPR-Cas plasmids to the cloning technology. In addition to the above technologies, *B. subtilis* gene-editing technology have been further developed based on CRISPR-Cas9 technology. Hao et al. (2020) design a CRISPR-Cpf1-based toolkit employing a type V Cas protein, Cpf1 from *F. novicida*. Using this platform, they precisely delete single gene and gene cluster in *B. subtilis* with high editing efficiency, such as *sacA, ganA, ligD,ligV*, and *bac* operon. Wu et al. (2020) engineer the *F. novicida* U112 CRISPR/Cpf1 system, a powerful tool called CAMERS-B is constructed for engineering *B. subtilis*, and a SOMACA (Synthetic Oligos Mediated Assembly of crRNA Array) method is constructed to build crRNA array. Liu et al. (2019) develop a CRISPR-Cas9n-based multiplex genome editing system for iterative genome editing in *B. subtilis*. This system enables to introduce various types of genomic modifications with more satisfying efficiency than using CRISPR-Cas9, especially in multiplex gene-editing. Through the use of CRISPR gene-editing technology, the production of plipastatins, riboflavin, amorphadiene (Ahmed-Hocine et al., 2020; Zou et al., 2020; Song et al., 2021) has been successfully increased in *B. subtilis*. Due to the high editing efficiency of the CRISPR-Cas9 cassette, CRISPR-Cas9 can be used to accurately overexpress single or multiple MK-7 pathway synthesis-related key genes or knock out related genes impeding Mk-7 synthesis. Thus, we believe that the CRISPR-Cas9 system will emerge as one of the most efficient gene-editing tools for the MK-7 anabolism pathway to enhance the MK-7 production from *B. subtilis*.

## CRISPRi (CRISPR Interference) and CRISPRa (CRISPR Activation) System

The dCas9 system is integral to CRISPR technology application. Currently, it is applied to regulate gene transcription, such as the inhibition and activation of transcription known as CRISPRi (CRISPR interference) and CRISPRa (CRISPR activation) system, respectively. Studies have shown that dCas9 mutate at the two enzyme active sites can stably bind to target DNA and inhibit the transcription process of *E. coli* genes by using its steric hindrance effect (Qi et al., 2013). Another example is the transcriptional repressor screening of the KRAB domain. The dCas9-KRAB fusion protein bounds near the transcription start site (TSS) of the gene. KRAB could effectively recruit histone modifiers to form heterochromatin, which inhibits gene transcription at the binding site resulting in the knockdown of target genes (Gilbert et al., 2013).

Locked nucleic acid (LNA), the first knockdown technology, is entirely based on the complementary pairing of two nucleic acid strands. However, compared with off-target genes such as RNAi, target genes knockout is less efficient, therefore produces false-positive data. Despite this shortcoming of target gene knockdown techniques, CRISPRi is the most effective technique because it does not interfere with non-target genes (Stojic et al., 2018). Furthermore, recent advancements in the design and construction of sgRNA promote the wide adoption of CRISPR technology in gene editing-related projects. Therefore, CRISPRi technology for gene knockdown has clear advantages and cost-effective than the traditional RNAi technology.

CRISPR emergence enables the activation of gene expression *in situ*. The earliest CRISPRa system use the same VP16 domain as the Tet-on/off system to activate gene transcription, but unlike the artificially design TRE promoter, the endogenous gene promoter is under complex regulation, even if ten copies of VP16 are used. This is because endogenous genes typically recruit proteins to form a huge complex when transcribed. The idea is that researchers can imitate this process, using Cas9 and sgRNA powerful transformation ability to enhance the gene transcription processes (Chavez et al., 2016).

VPR, SAM, and Suntag technologies are the most common Cas9 activator designs, playing synergistic roles together. Firstly, VPR technology enhances the ability of the fusion protein to activate gene transcriptions (Chavez et al., 2015). While SAM (synergistic activation mediator) technology utilizes sgRNA to transform and add MS2 sequence to the two neck loop structures of sgRNA for MCP protein recruitment, then fuse the MCP protein with P65-hsf1 domains and dCas9. Whereas the Suntag technology uses the principle of antigen and antibody. The long-chain antigen here fused to dCas9 recruit VP64 carried by multiple antibody scFv at a time, amplifying its activation effect (Tanenbaum et al., 2014). These second-generation activator effects are significantly better than the earliest VP64 standard with no significant off-target phenomenon (Chavez et al., 2016), making them good candidates to induce endogenous gene transcriptions.

CRISPR knockout abilities such as high efficiency, precision, and low off-target effects characterize both the CRISPRi and CRISPRa systems. Although some of these molecules are complicated in design when acting on different target genes; however, the production cost is relatively less expensive, making them suitable for constructing a full-gene suppression or activation library. Lu et al. (2019) determine the optimal targeting window for CRISPRa and CRISPRi, which further proved that the system could be used as a single master regulator by designing location-specific gRNAs to activate or inhibit the expression of different genes simultaneously. They develop a new CRISPR-assisted Oligonucleotide Annealing-based Promoter Shuffling (OAPS) strategy, which can screen strong promoters to increase rate-limiting enzyme expressions and relieve feedback inhibition for pathway optimization. Therefore, in regards to biosynthetic pathway application, the OAPS combined with dCas9-mediated multi-directional transcription program and OAPS to increase *B. subtilis* amylase BLA production by 260∼fold. Their results further prove that CRISPRi and CRISPRa systems are highly effective and can be applied for single gene knockdown or activation and whole gene functional screening. i.e., *B. subtilis* pathway bioengineering. A prominent application of CRISPR interference in bioengineering is experimented by Peters et al. (2016), where they knock down every essential gene in *B. subtilis* to probe their phenotypes, and their data provide an integral systematic research framework for future purposes. Moreover, their framework applies to *in-vivo* probing of essential gene modifications across diverse microorganisms, and it is flexible for comparative analysis studies. Similarly, Dong et al. (2020) induce the CRISPRi system by xylose to further increase the production of lactate-n-neotetraose (LNnT). Hence, We hypothesis that the CRISPR-dCas9 system will become one of the most effective gene-editing tools in the MK-7 anabolic pathway.

Different functional elements could facilitate dCas9/sgRNA manipulations for genome sequencing, such as gene transcription, epigenetic modification, and genome mapping imaging (Xu and Qi., 2019). With this condition, CRISPR-dCas9 can resolve off-target effects and will be effectively applicable to multi-gene editing. Several reports on the CRISPR-Cas9 gene-editing of *B. subtilis* have proven the feasibility of CRISPR gene-editing applications in *B. subtilis*. The expression of MK-7 by *B. subtilis* is related to multi-gene regulation. At the moment, obtaining high-yielding MK-7 strains involves activating several MK-7 associated genes or inhibiting the expression of branch genes. For this research gap, It is necessary to develop more diverse CRISPR gene-editing methods (such as CRISPRa and CRISPRi) to achieve efficient high yielding MK-7 productions. Thus, we suggest that CRISPR-dCas9 would enhance the *B. subtilis* to transform into high-yielding MK-7 strains.

## Concluding Remarks

**FIGURE 3:**
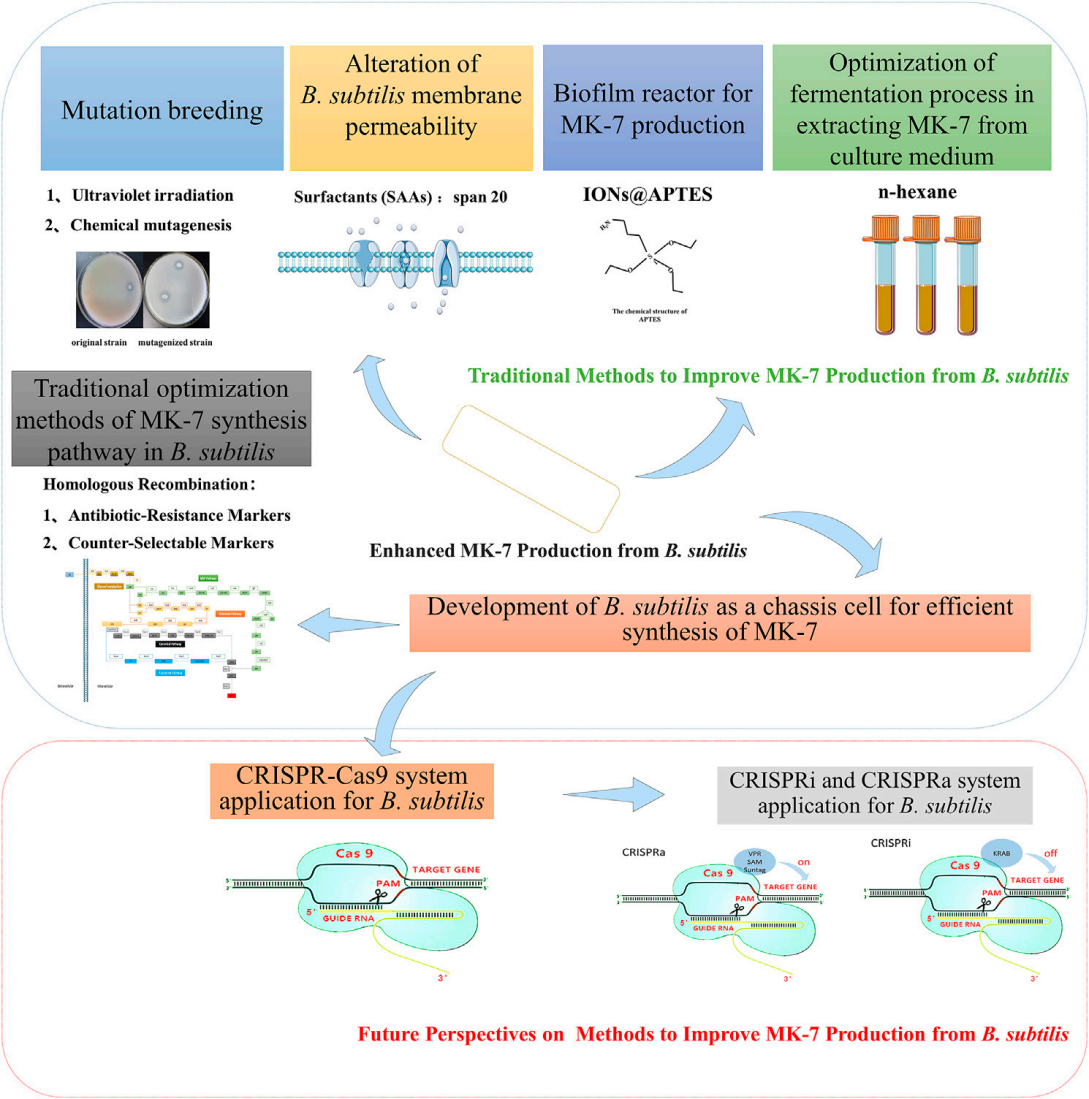
Methods to enhance MK-7 production from *B. subtilis*.

MK-7 has demonstrated beneficial effects on several disorders in humans. including bone metabolism and blood clotting. For these reasons, there are desires to improve the MK-7 production techniques. The conventional approaches include chemical synthesis and mutagenesis breeding of MK-7-requiring bacteria to enhance MK-7 production from *B. subtilis*. These methods have many drawbacks, especially high production costs and time-consuming operations. Genetically improved Gram-positive bacteria, such as *B. subtilis*, have gained increasing attention for the MK-7 biosynthesis. This review has addressed the possible advanced techniques relevant for MK-7 production from *B. subtilis.* Specifically, the application of CRISPR-Cas9 in *B. subtilis* is introduced in this article. It described the advantages of CRISPRi (CRISPR interference) and CRISPRa (CRISPR activation) systems and their potentials to becoming one of the most effective gene-editing tools for MK-7 anabolic pathways (Figure 3). The role of CRISPR-dCas9 to develop *B. subtilis* as a chassis is a good reference for future possibilities.
